# Effect of a new incision management system (PREVENA®) on wound healing after endophlebectomy of the common femoral vein: a case series

**DOI:** 10.1186/s13256-016-0930-7

**Published:** 2016-05-27

**Authors:** A. Gombert, M. E. Barbati, C. Wittens, J. Grommes, H. Jalaie

**Affiliations:** 1 European Vascular Center Aachen-Maastricht, Department of Vascular Surgery, University Hospital RWTH Aachen, Pauwelsstraße 30, 52074 Aachen, Germany; 2 European Vascular Center Aachen-Maastricht, Department of Vascular Surgery, University Hospital Maastricht, Maastricht, Netherlands

**Keywords:** Post-thrombotic syndrome, Endophlebectomy, Lymph fistula, Wound infection, Incision management system, PREVENA®

## Abstract

**Background:**

New endovascular techniques facilitate treatment of complex deep vein obstructions in cases of post-thrombotic syndrome. In a relevant number of these patients, endophlebectomy including the implantation of an arteriovenous fistula between the common femoral artery and the femoral vein is indispensable in order to establish a good inflow. These procedures display a high risk of wound complications. Despite conservative efforts to prevent these postoperative complications, wound healing problems occur in more than 20 % of cases. The present case report is the first description of wound dressing using a PREVENA® incision management system in cases of endophlebectomy.

**Case presentation:**

A single center’s experience with the incision management system PREVENA®, which was used after endophlebectomy and venous stenting in complex hybrid procedures in three white men aged 46 years, 53 years, and 61 years is the subject of this case report. Although the surgical procedures were performed under therapeutical anticoagulation and took a couple of hours, no wound complications occurred.

**Conclusions:**

These encouraging results underline a potential benefit of the incision management system PREVENA® in cases of complex venous recanalization including endophlebectomy of the femoral vein as well as the implantation of an arteriovenous fistula.

## Background

Impaired wound healing may be attributed to defects in the normal tissue as response to injury and to poor wound management. Edema, ischemia, existent systemic infection, diabetes, smoking, as well as peripheral artery disease or venous insufficiencies are risk factors for poor wound healing and wound complications. Furthermore, these are associated with poor surgical outcome.

The majority of vascular surgery procedures are classified as clean surgery by the US National Research Council because they are performed in uninfected tissue without signs of inflammation [1]. The reported incidence of surgical wound infections after lower limb vascular surgery varies between 3.5 and 32 % [1–4]. Therapy for symptomatic chronic venous obstruction and post-thrombotic syndrome (PTS) has changed during the last few years. Standard conservative treatments including anticoagulation, compression therapy, and mobilization are challenged by endovascular recanalization, partly supplemented by open surgical reconstruction. These new methods deliver good patency rates and an improved quality of life [5–7]. If these post-thrombotic changes of the venous vessels, which may involve the iliac as well as the femoral vessels, additionally affect the common femoral vein (CFV), an endophlebectomy has to be performed to recanalize this segment [7, 8]; otherwise the impaired inflow would hamper the patency of the recanalization. Furthermore, the still remaining venous hypertension in the distal part of the lower extremity would not allow complete improvement of complaints.

Although patients with PTS receiving interventional or surgical treatment tend to be young and healthy, the rate of wound complications and lymph fistula after endophlebectomy is high [9]. Data that address complications after endophlebectomy are scarce [7]. Vogel *et al*. described three cases with severe complications that needed additional therapy due to thrombosis and hematoma after venous surgery [6]. One of their patients had to be treated with surgical reintervention including wound debridement and vacuum therapy. Both pre-existing venous outflow obstruction and arteriovenous fistula (AVF), which has to be implanted during recanalization, increase the risk of lymphatic leakage. The normal amount of fluid expelled from the leg through the venous system is approximately 90 %; the amount expelled by the lymphatic system is approximately 10 %, but under post-thrombotic circumstances the amount can be more than 20 %.

Recent studies, which compared the outcome of an incision management system (IMS) with conventional wound dressings in cases of groin incision, showed 20 % less groin infections in patients with peripheral artery disease (PAD) [10]. In contrast to negative pressure wound therapy, which is established in the clinical routine and scientifically evaluated in different areas of application, an IMS has to be seen as a prophylaxis to avoid wound healing complications [11, 12].

Animal testing with Yucatan swine revealed improved mechanical properties and better wound conditions after the use of an IMS in comparison with standard care wound therapy [13]. The lateral tensile stress of closed incisions can be decreased by approximately 50 % [14]. Correspondingly it could be shown that the rate of hematoma and seroma of surgical incision sites can be reduced compared with standard wound dressings in animal testing. A reduced upregulation of genes associated with systemic inflammation, hypoxemia, and impaired wound healing was recognized within the first week of use [13].

In the available literature, no case of IMS-related complication such as skin lesions could be detected [15, 16].

Based on these findings, this study was established to examine the benefit of IMS use in cases of venous reconstruction: A reduced wound healing complication rate should be demonstrated. Our patients are three men aged 53 years, 46 years, and 61 years who have PTS due to a deep vein thrombosis (DVT) of the lower extremity.

## Case presentation

### Recanalization of obliterated femoral and iliac veins

After the standardized diagnostic work-up, including duplex ultrasound (DUS) and magnetic resonance venography (MRV), the surgical procedure took place in a dedicated intervention room or a dedicated vascular surgical suite under general anesthesia. Fluoroscopic assistance is necessary to enable a combined approach with endophlebectomy of the CFV with or without AVF and interventional disobliteration of the iliac veins [17].

The procedure starts with cannulation of the popliteal vein in prone position or the femoral vein (FV) in supine position under ultrasound guidance. Furthermore, an antegrade angiography shows the exact localization, anatomy, and extent of the obstruction. Sometimes a retrograde cannulation via the jugular veins is necessary to perform the intervention. While using conventional guidewires (including stiff wires) and catheter techniques, it is usually possible to cross an occluded vein. After dilatation of the involved areas of the iliac veins, self-expandable venous stents are placed. These stents, which are provided by different manufacturers, have to fulfil specific demands for the venous system. The focus on radial force and flexibility is the main difference between arterial stents. Further dilatations and a venography of the obstruction level of the FV are crucial. If the inflow is poor, an endophlebectomy including patch reconstruction of the CFV has to be performed. As a standard, a bovine patch is used. This may include the installation of an AVF between the FV and artery by using a 6-mm ringed polytetrafluoroethylene (PTFE) prosthesis. Due to high pressure and the experience of the performing vascular surgery department, the use of an autologous vein should be avoided considering the risk of pseudoaneurysm formation in this setting. Furthermore an interventional closure of the fistula, which usually will be performed 3 to 6 months after the venous recanalization under local anesthesia, will be more sophisticated. Wound closure was performed using intracutaneous, absorbable suture material.

After the surgical procedure, which was performed under therapeutical anticoagulation, therapy with anticoagulants is continued for a minimum of 6 months. Low molecular weight (LMW) heparin, phenprocoumon, or rivaroxaban can be used. In case of phenprocoumon the target international normalized ratio (INR) is 2.5 to 3.5.

Follow-up includes DUS examinations, clinical evaluation, and, if necessary, X-ray examination of the pelvic region to detect a dislocation or obstruction of the stent prosthesis.

### Wound therapy using an IMS (PREVENA®)

According to the instructions for use, the IMS PREVENA® (Acelity, San Antonio, Tx, USA) allows the management of primarily closed surgical incisions. The system includes a dressing with an integrated drape, a foam bolster, and a skin-contacting interface layer for ease of application. After placement of the IMS on a closed surgical incision a negative pressure of 125 mmHg is applied continuously to the wound for 5 to –7 days without removal of the system [18]. In the cases presented in this case report, a 7-day treatment was chosen. The acceptance of the IMS is high due to the absence of noise during therapy and the non-limited mobility of the patients. After 7 days, the IMS was removed, an examination of the wound, including photo-documentation, was performed. The same procedure was done on the 15th and the 30th day after treatment.

### Case 1

**Fig. 1:**
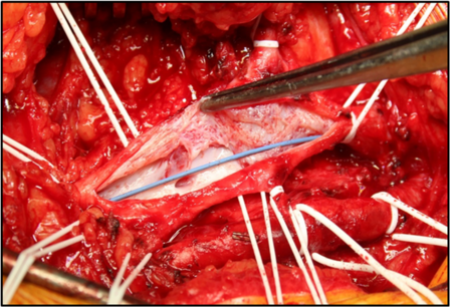
Intraoperative picture of the post-thrombotic changes of the common femoral vein before and after endophlebectomy

**Fig. 2:**
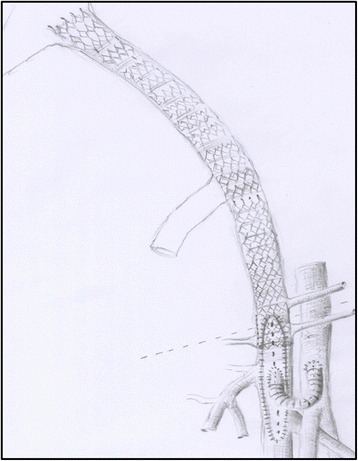
Picture of a venous reconstruction including venous stenting of the iliac veins, endophlebectomy, bovine patch of the common femoral vein, and arteriovenous fistula between the common femoral vein and common femoral artery

The first patient, a 53-year-old white man, with a body mass index (BMI) of 27.5, had PTS after DVT of his right femoral and iliac vein. His medical history included arterial hypertension and tobacco smoking. His medication included antihypertensive drugs. After multiple surgical procedures in September 2013, due to infections related to resection of his prepatellar bursa, he developed a DVT, involving his external iliac vein (EIV) and FV. A computed tomography (CT)-angiography and phlebography, performed in April 2014, showed post-thrombotic obliteration of the proximal part of his great saphenous vein (GSV), his FV and his EIV. His common iliac vein (CIV) and inferior vena cava (IVC) were free of post-thrombotic changes. Despite appropriate medical treatment with the anticoagulant rivaroxaban (Xarelto®) and compression stockings, he had venous claudication, severe pain, and chronic swelling of his right leg. Therefore a recanalization of his EIV, combined with an endophlebectomy of his CFV and an AVF from his femoral artery to his CFV, was planned. The venotomy was reconstructed with a bovine patch (see Figs. 1, 2). The recanalization of the distal part of his CIV and EIV was completed using two sinus–Venous stents® (12×80 mm, 14×100 mm; Optimed, Ettlingen, Germany). The operation took 663 minutes under full anticoagulation (activated clotting time 200 seconds). After the positioning of a drain tube and wound closure, the PREVENA® IMS was applied.

**Fig. 3:**
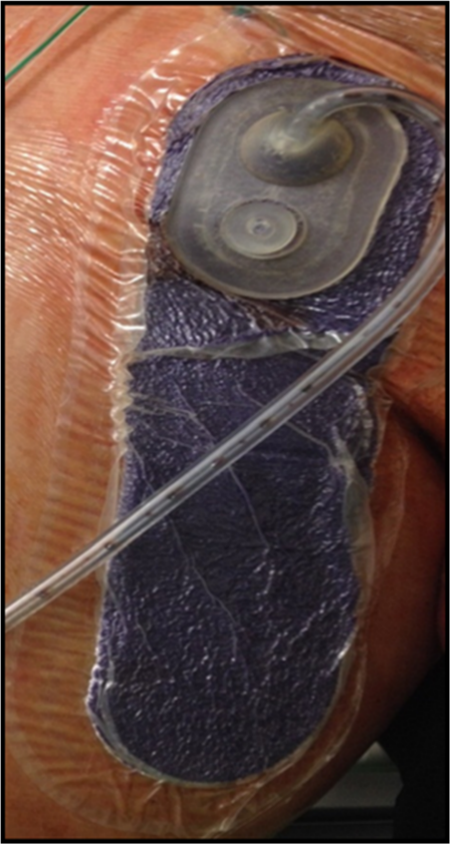
Postoperative application of an incision management system (Case 1)

The drain tube secreted 2200 ml of mainly lymphatic fluid and the hemoglobin dropped slightly from 14.0 to 11.9 g/dl within 72 hours. After removal of the drain from his groin incision, the IMS remained until the seventh day after surgery without any problems (see Fig. 3).

**Fig. 4:**
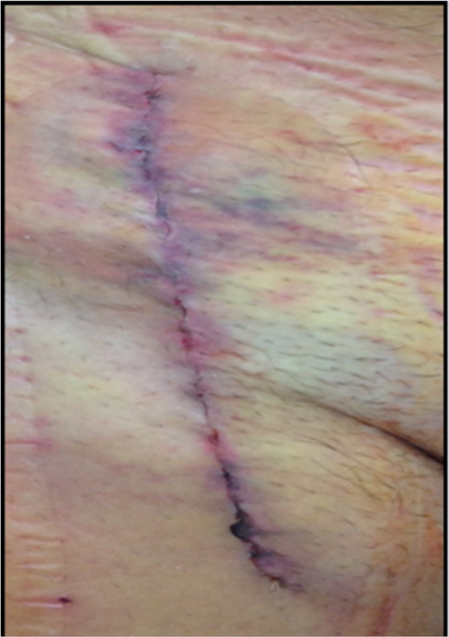
Wound conditions after removal of the PREVENA® incision management system (Case 1)

Pneumatic compression of his right leg was performed continuously for 48 hours. Mobilization was started 48 hours after the surgical treatment. After 7 days the PREVENA® IMS was removed without the need of replacement. No wound complications were observed (see Fig. 4). An ultrasound examination showed a patent venous reconstruction.

**Fig. 5:**
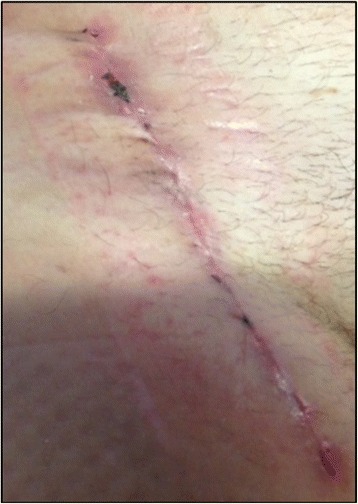
Wound conditions 30 days after removal of the PREVENA® incision management system (Case 1)

Identical findings were collected on the 15th and the 30th day after surgery: There were no further signs of wound complications and the reconstruction remained patent (see Fig. 5).

### Case 2

The second case was a 61-year-old white man, BMI of 28.5, who had venous claudication and severe pain in his left leg for more than 1 year due to an old thrombosis of his EIV. His medical history showed arterial hypertension, dyslipidemia, and coronary heart disease. His medication included statins, antihypertensive drugs, and aspirin. After magnetic resonance (MR)-phlebography, which confirmed the diagnosis, the venous reconstruction was took place: A recanalization of his EIV combined with an endophlebectomy of his CFV and an AVF from his femoral artery to his CFV was realized. A bovine patch was used for the reconstruction of the venotomy; the recanalization of the distal part of his CIV and EIV was completed using two sinus–Venous stents® (12×80 mm, 14×100 mm; Optimed, Ettlingen, Germany). The operation took 320 minutes under full anticoagulation and was finished by positioning the drain tube and application of the PREVENA® IMS. The drain tube secreted 450 ml of mainly lymphatic fluid; no relevant hemoglobin decrease could be recognized.

**Fig. 6:**
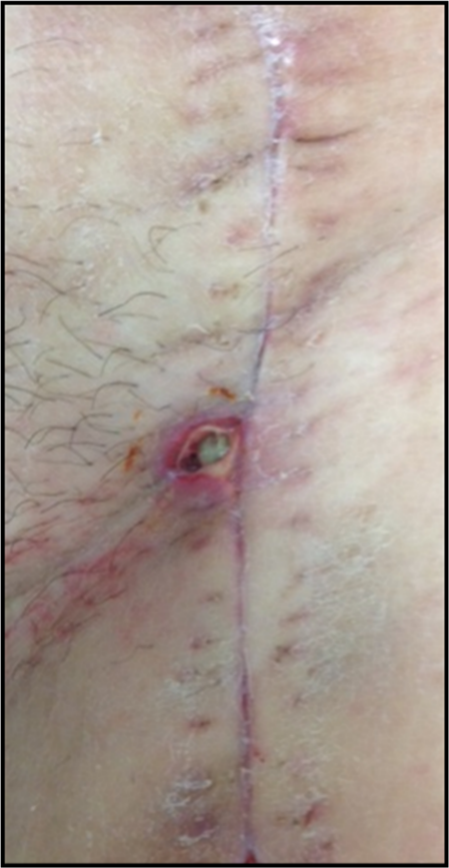
Szilagyi I° classified wound complication 15th postoperative day (Case 2)

The length of his hospital stay after venous reconstructive surgery was 7 days and he was discharged after removal of the IMS without any wound healing disorders. In the first postoperative follow-up (15 days after surgery), a small 0.5×0.5 cm wound healing disorder in the median incision was observed; it was classified as Szilagyi I°. With conservative wound dressing, the wound healed within the next 14 days without any subsequent problems (see Fig. 6).

### Case 3

**Fig. 7:**
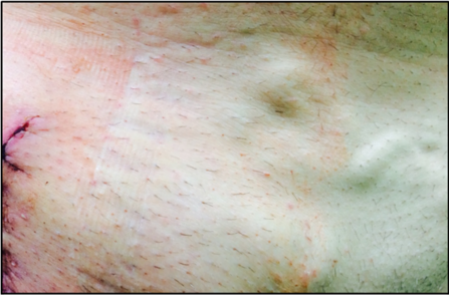
Incision in the right groin after venous reconstruction and venous collateralization in the lower abdominal area can be seen. In addition the imprint of the incision management system can be seen around the incision (Case 3)

The third case, a 46-year-old white man, BMI of 29, showed a history of DVT of his right leg in 1995. His medical history showed arterial hypertension, obesity, tobacco smoking, dyslipidemia, and coronary heart disease. His medication included statins, antihypertensive drugs, and aspirin. After a failed thrombectomy within the same year, he had venous claudication, chronic swelling, and chronic ulceration of his lower leg for nearly 20 years. Hence he was referred to our department in 2014. An ultrasound and MR-phlebography detected strong venous collateralization in his abdominal area (see Fig. 7). Treatment was performed with endophlebectomy of his CFV and creation of an AVF, as well as venous stenting including his EIV, his CIV, and his IVC realized by three venous stents. Wound dressing was performed by the use of an IMS in the operating theatre. After 7 days, the dressing was removed and no further dressing was necessary. During the 6-months follow-up until the interventional closure of the AVF, no wound complications occurred.

## Discussion

Although the techniques of the open surgical and the endovascular approach of chronic venous obstruction have developed within the last few years, the urgent problem of wound complications, such as lymphatic fistula, remains unsolved. These complications are associated with extended hospital stay, a higher rate of wound infections, as well as pain, the use of antibiotics, surgical re-interventions, and failure of the venous reconstruction. Moreover, the increased in-hospital costs as well as the decreased quality of life due to an elongated stay in hospital with long periods of immobilization as a part of the lymph fistula treatment should be mentioned.

The recruitment of lymphatic vessels due to the venous obliteration in patients with PTS is one cause for the wound complications which may occur after surgical incision. In this setting, the necessity of therapeutic anticoagulation contributes to the formation of a hematoma.

Furthermore, the AVF increases the venous flow and the local pressure conditions of the venous system to non-physiological levels. The high rate of surgical complications, such as wound infections, venous recanalization failure, and even graft infection, is challenging. Medical treatment for weeks, chronic pain, as well as surgical and endovascular re-interventions, may be the consequences of wound complications related to this hybrid procedure.

## Conclusions

The presented cases illustrate the potential benefit of an IMS such as Prevena® after a complex venous hybrid recanalization including endophlebectomy of the CFV and stent angioplasty of the femoral and iliac veins.

To evaluate this improved surgical site treatment with reduced wound complication rates, further, randomized, multicenter studies are necessary.

## Abbreviations

AVF: arteriovenous fistula
BMI: body mass index
CFV: common femoral vein
CIV: common iliac vein
CT: computed tomography
DUS: duplex ultrasound
DVT: deep vein thrombosis
EIV: external iliac vein
FV: femoral vein
GSV: great saphenous vein
IMS: incision management system
INR: international normalized ratio
IVC: inferior vena cava
LMW: low molecular weight
MR: magnetic resonance
MRV: magnetic resonance venography
PAD: peripheral artery disease
PTFE: polytetrafluoroethylene
PTS: post-thrombotic syndrome
